# Development of Cu_3_N electrocatalyst for hydrogen evolution reaction in alkaline medium

**DOI:** 10.1038/s41598-022-05953-x

**Published:** 2022-02-07

**Authors:** Aparna Sajeev, Aleena Mary Paul, Ravi Nivetha, Kannan Gothandapani, Tamil Selvi Gopal, George Jacob, Muthumareeswaran Muthuramamoorty, Saravanan Pandiaraj, Abdullah Alodhayb, Soo Young Kim, Quyet Van Le, Pau Loke Show, Soon Kwan Jeong, Andrews Nirmala Grace

**Affiliations:** 1 Centre for Nanotechnology Research, Vellore Institute of Technology, Vellore, India; 2 College of Science, King Saud University, P. O. Box 2455, Riyadh, 11451 Saudi Arabia; 3 Department of Self Development Skills, CFY Deanship, King Saud University, Riyadh, Saudi Arabia; 4 Department of Physics and Astronomy, College of Science, King Saud University, P. O. Box 2455, Riyadh, 11451 Saudi Arabia; 5 Department of Materials Science and Engineering, Institute of Green Manufacturing Technology, Korea University, 145, Anam-ro Seongbuk-gu, Seoul, 02841 South Korea; 6 Institute of Research and Development, Duy Tan University, Da Nang, 550000 Vietnam; 7 Department of Chemical and Environmental Engineering, Faculty of Science and Engineering, University of Nottingham Malaysia, Jalan Broga, 43500 Semenyih, Selangor Darul Ehsan Malaysia; 8 Climate Change Technology Research Division, Korea Institute of Energy Research, Yuseong-gu, Daejeon, 305-343 South Korea

**Keywords:** Energy science and technology, Materials science, Nanoscience and technology

## Abstract

A wide variety of electrocatalysts has been evolved for hydrogen evolution reaction (HER) and it is reasonable to carry out HER with low cost electrocatalyst and a good efficiency. In this study, Cu_3_N was synthesized by nitridation of Cu_2_O and further utilized as an electrocatalyst towards HER. The developed Cu_3_N electrocatalyst was tested and results showed a low overpotential and moderate Tafel slope value (overpotential: 149.18 mV and Tafel slope 63.28 mV/dec at 10 mA/cm^2^) in alkaline medium with a charge transfer resistance value as calculated from electrochemical impendence spectroscopy being 1.44 Ω. Further from the experimental results, it was observed that the reaction kinetics was governed by Volmer–Heyrovsky mechanism. Moreover, Cu_3_N has shown an improved rate of electron transfer and enhanced accessible active sites, due to its structural properties and electrical conductivity. Thus the overall results show an excellent electrochemical performance, leading to a new pathway for the synthesis of low cost electrocatalyst for energy conversion and storage.

## Introduction

With the depletion of fossil fuels and an increasing threat of global warming and environmental pollution, there is a huge quantum of research in the development of new energy resources^1–3^. Of the various important fuels, hydrogen is an excellent alternative as it is clean with CO_2_ neutral, having high gravimetric energy density and eco-friendly green renewable energy source^4–6^. The diverse techniques for the production of H_2_ include electrochemical and photoelectrochemical water splitting, thermolysis, biomass pyrolysis, hydrocarbon steam reforming, and coal gasification^7–10^. Among these techniques, electrochemical hydrogen evolution reaction (HER) is a simple and an efficient technique to meet the future energy demand. The cathodic HER involves 2e^−^ transfer process with multi-step reaction consisting of absorption, reduction and desorption.

1 $${2H}^{+ }+ {2e}^{- }\to {H}_{2ds}.$$

The initial step is Volmer reaction (Eq. (2)) in which coupling of protons with electron occurs at the surface of catalysts forming adsorbed hydrogen atom. Then, adsorbed hydrogen atom reacts with another proton from solution in conjunction with electron transfer to form hydrogen molecule via Heyrovsky reaction (Eq. (3)). In the final step, two adsorbed hydrogen atoms combine to form hydrogen molecule—Tafel reaction^11,12^ (Eq. (4)), where

2 $${H}^{+ }+ {e}^{- }\to {H}_{ads},$$

3 $${2H}_{ads}+{H}^{+ }+ {\text{e}}^{-}\to {H}_{2},$$

4 $${2H}_{ads}\to {H}_{2}.$$

In general, platinum is the ideal catalyst for HER with the desired characteristics such as low onset potential, Tafel slope and high durability but the high cost and scarcity hampered its large scale application in hydrogen production^13–15^. Thus, it is crucial to develop abundant and highly efficient electrocatalysts for large scale hydrogen production. Over the past few decades, research is focused on developing first row transition metals as efficient electrocatalyst for HER^16^. Copper is a promising catalyst and similar analogue to Pt metal, but has a limited activity towards HER due to its deficiency in the capture of H atom^17–21^. Numerous efforts have been taken to synthesize copper with transition metal sulphides, carbides, phosphides and dichalcogenides to overcome this issue for improving HER performance^22–29^. Copper Nitride (Cu_3_N) is a metastable semiconductor that has been proposed as efficient cathodic materials for energy conversion and storage applications, because of their unique physiochemical optical, electrical and its thermal properties^30–32^. Cu_3_N has drawn attention in other fields like optical device storage, fuel cells, high-speed ICs, metallic microscopic links, CO_2_ reduction, energy storage and energy production^30–32^. Various routes have been explored for the reduction of particle size and different morphology of Cu_3_N. For instance, Pereira et al. prepared Cu_3_N from CuF_2_ at 300 °C in NH_3_ atmosphere. XRD measurements revealed dark green power of Cu_3_N without any trace of oxidation or residual CuF and TEM images exhibited nanodomains of Cu_3_N materials. The obtained Cu_3_N were used as negative electrode for lithium battery application^33^. In recent years, Cu_3_N in the form of thin films have been mainly synthesized by molecular beam epitaxy (MBE), radio frequency (RF), active laser deposition (ALD), ion assisted deposition, ultrasonic plasma spray method and magnetron sputter ion plating. Other preparation method for Cu_3_N particles includes solvothermal and ammonolysis method^32,34^. Deshmukh and co-workers reported the synthesis of ultra-small Cu_3_N nanoparticles via one step reaction between copper (II) methoxide and benzylamine. TEM imaged confirmed that Cu_3_N has ultra-small particle morphology with ~ 2 nm thickness. These Cu_3_N nanoparticles provided pathways for the development of efficient cathode materials to enhance lithium ion batteries application^35^. Here in, Cu_3_N nanoparticles have been explored as an efficient electrocatalyst for electrochemical hydrogen evolution reaction. The prepared Cu_3_N material as electrocatalyst possesses intrinsic HER activity, which might be related to their electronic structure and oxidation state of Cu, resulting in Cu^+^ increasing the electrochemically active surface to enhance hydrogen evolution performance. In this work Cu_3_N nanoparticle were synthesized from nitridation of Cu_2_O and to further confirm the formation and morphology, various investigations were done like XRD, FTIR, SEM and BET measurements. Cu_3_N as electrocatalyst exhibited a considerable catalytic performance of HER in alkaline electrolyte, a reasonable current density of 10 mAcm^−2^ at an overpotential of 149.18 mV. The good HER performance might owe to the large surface area and favourable electrical conductivity of Cu_3_N particles.

## Experimental

All the chemicals and reagents used were of analytical grade and used without any further purification. Copper (II) sulphate pentahydrate (CuSO_4_·5H_2_O), Sodium hydroxide (NaOH), l-Ascorbic acid (C_6_H_8_O_6_) were purchased from Sigma-Aldrich Chemicals Pvt. Ltd. and double distilled water was used in the synthesis by using Milli-Q water.

### Preparation of Cu_2_O nanoparticles

**Figure 1:**
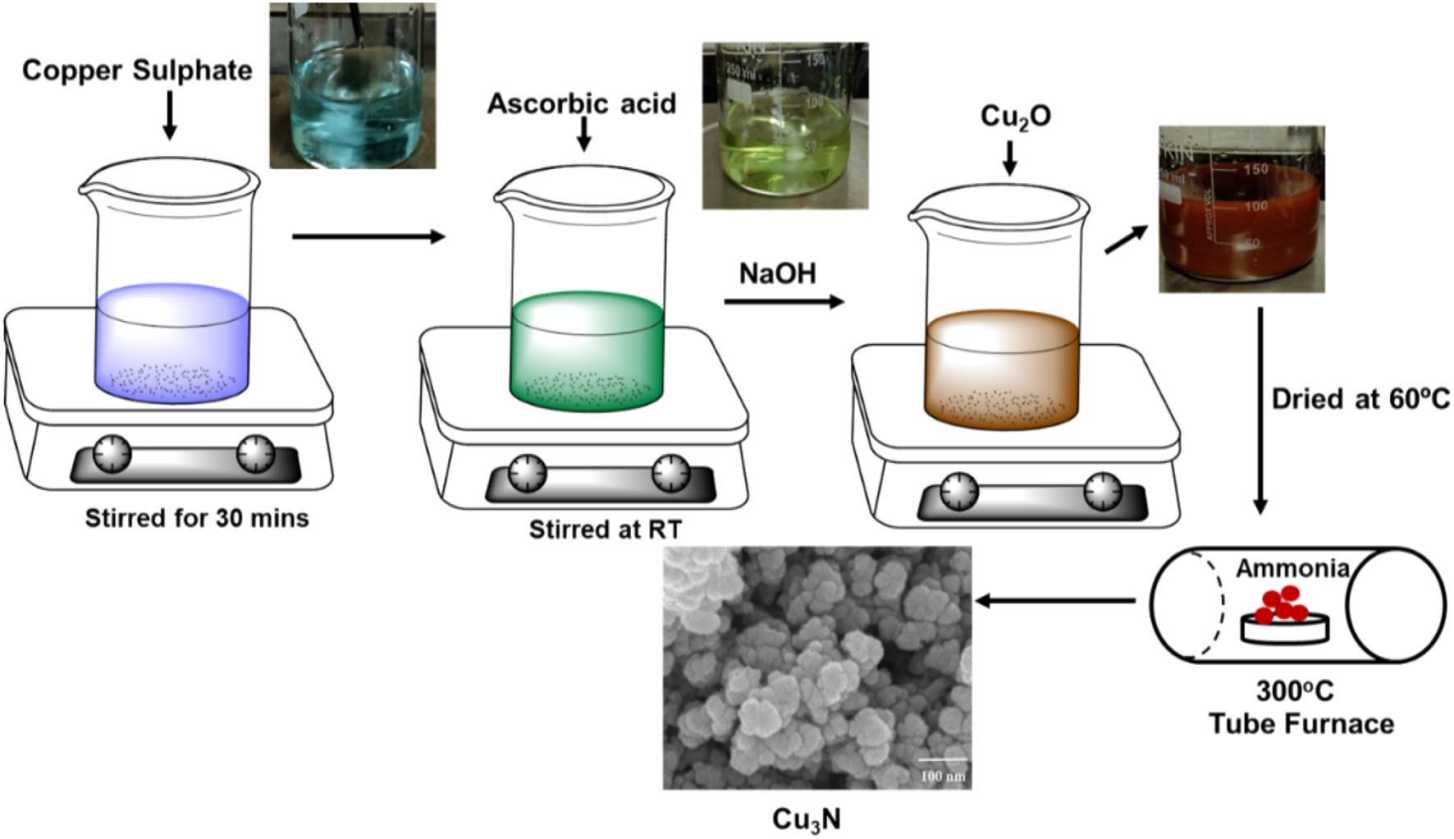
A schematic of the preparation of Cu_2_O and Cu_3_N nanoparticles.

The synthesis procedure of cuprous oxide (Cu_2_O) was adopted from the previous literature report^36^ with slight modifications. Typically, 2 mmol of copper sulphate solution was dissolved in 50 ml of DI water and simultaneously 20 mmol of NaOH was added drop wise into the mixture. Then the mixture was continuously stirred at ambient temperature. Later, a capping agent of 4 mmol ascorbic acid was added into the above solution. Finally the reaction mixture was stirred continuously, stirred for 30 min at ambient temperature. The resultant product turns the solution to brick red colour as given in Fig. 1, which indicated the formation of cuprous oxide (Cu_2_O) nanoparticles. Further the obtained Cu_2_O nanoparticles were washed with DI water and ethanol for several times and dried at 60 °C for 12 h in vacuum oven.

### Preparation of Cu_3_N nanoparticles

The Cu_3_N nanoparticles were prepared via nitridation process of Cu_2_O^37^. Briefly, Cu_2_O nanoparticles was kept in an alumina tube and placed inside a furnace, which was subsequently heated under purified argon at 30 min. The tubular furnace was heated at a temperature of 250 °C for 21 h under ammonia atmosphere. The flow rate of ammonia gas was 60 ml/min for 1.5 h and the product was isolated by centrifugation (7500 rpm for 10 min). The resultant product was transferred into a petri dish, dried at 80 °C for 12 h. Further, Cu_2_O nanoparticles were heated with NH_3_ gas of different concentrations at different temperature, which is labelled as Cu_3_N-300/120 ml/min, Cu_3_N-300/160 ml/min and Cu_3_N-250/60 ml/min.

### Mechanism of Cu_3_N formation

Copper sulphate (CuSO_4_) reacts with NaOH solution in the reaction to form copper hydroxide Cu(OH)_2_. Then ascorbic acid as surfactant was added into copper hydroxide solution leading to the formation of copper oxides (Cu_2_O)^38^. In the last step, Cu_2_O powder was heated in NH_3_ atmosphere, which reacts with Cu_2_O to form Cu_3_N. The reaction mechanism for the formation of Cu_2_O and Cu_3_N is given below:

5 $$2 Cu{SO}_{4}+2 NaOH\to 2Cu\left(OH\right)+2 NaS{O}_{4},$$

6 $$2\text{Cu}\left(\text{OH}\right) \to {Cu}_{2}\text{O }+ {H}_{2}\text{O}.$$

After nitridation of Cu_2_O to Cu_3_N,

7 $${3\text{Cu}}_{2}\text{O}+ 2{\text{NH}}_{3} \to 2{\text{Cu}}_{3}N+ 3{\text{H}}_{2}\text{O}.$$

### Material characterization

The crystalline structure and phase identification of the synthesized material was characterized by Rigaku Miniflex Powder X-ray diffraction technique equipped with Cu-Kα (λ = 1.546 Å) over 2θ range of 10°–50°. The size and morphology of the as synthesized material were analysed using FESEM (Hitachi S-4800). Thermo gravimetric analysis (TGA) was performed in an air atmosphere with an SDT Q600 (TA Instruments).

### Preparation of electrodes

The glassy carbon electrode having a geometrical surface area of 0.07 cm^2^ was first polished with alumina slurry of 0.05 micron, followed by rinsing it with DI water, ethanol and acetone. The working electrode was prepared from 5 mg of Cu_3_N catalyst dissolved in 250 µl of ethanol. Later, 5 µl of the catalyst/5 µl of Nafion was pipetted with micro syringe and coated on cleaned glassy carbon electrode (GCE) surface using drop casting method. The coated electrode was then dried at room temperature for 12 h. Electrochemical testing was carried out by CHI 660C electrochemical workstation. Cyclic voltammetry, linear sweep voltammetry, Tafel plot and electrochemical impedance spectroscopy techniques were done to evaluate HER performance.

## Results and discussion

### Structure and morphology

**Figure 2:**
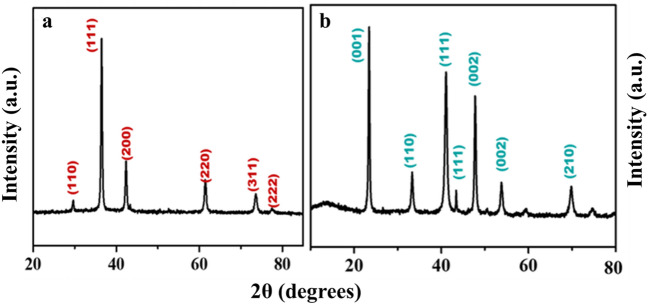
X-ray diffraction pattern of (**a**) Cu_2_O nanoparticle and the as synthesized, (**b**) Cu_3_N at 300 °C/160 ml/min ammonia flow rate.

**Figure 3:**
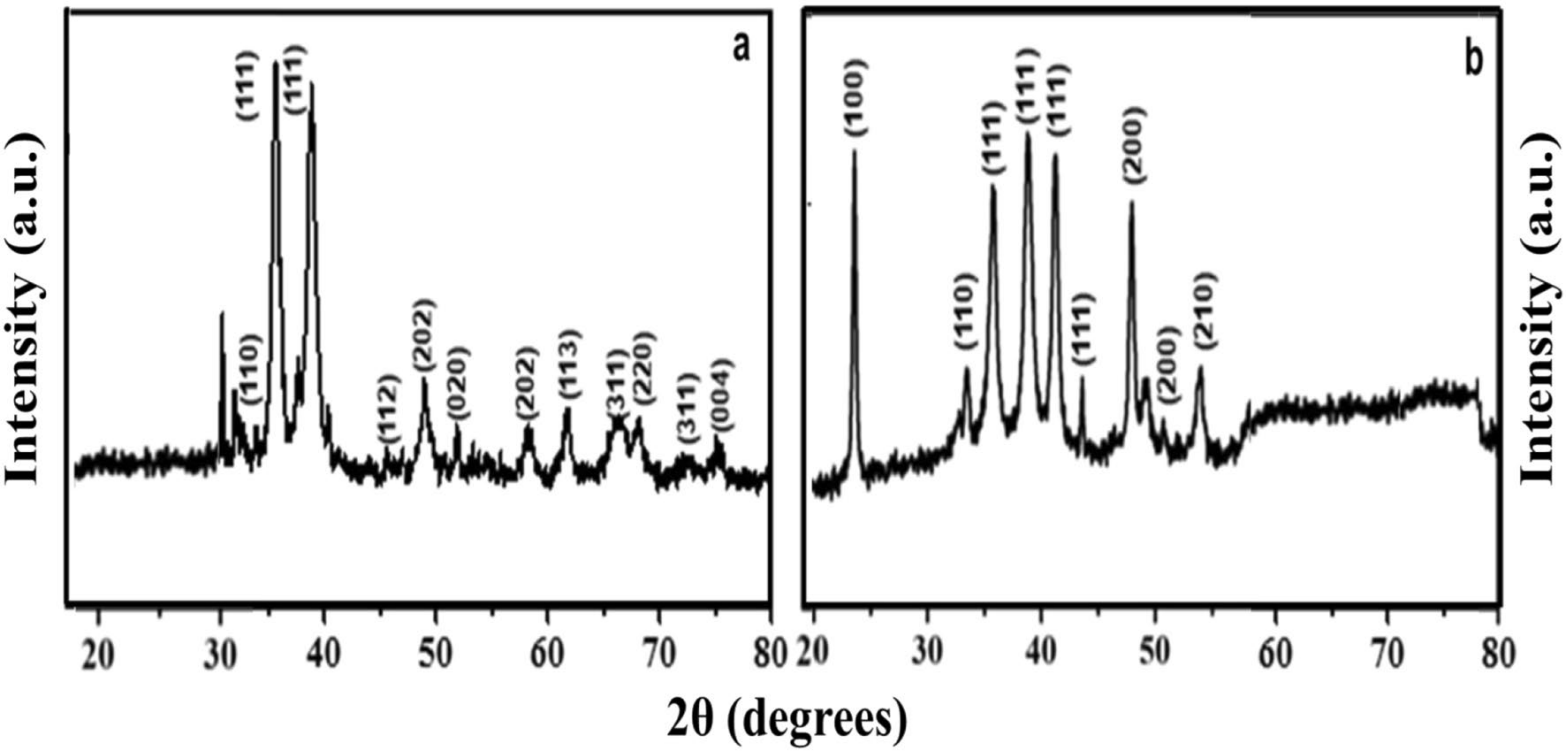
X-ray diffraction spectrum of the synthesized Cu_3_N nanoparticles at (**a**) 60 ml/min and (**b**) 120 ml/min ammonia flow rate.

**Figure 4:**
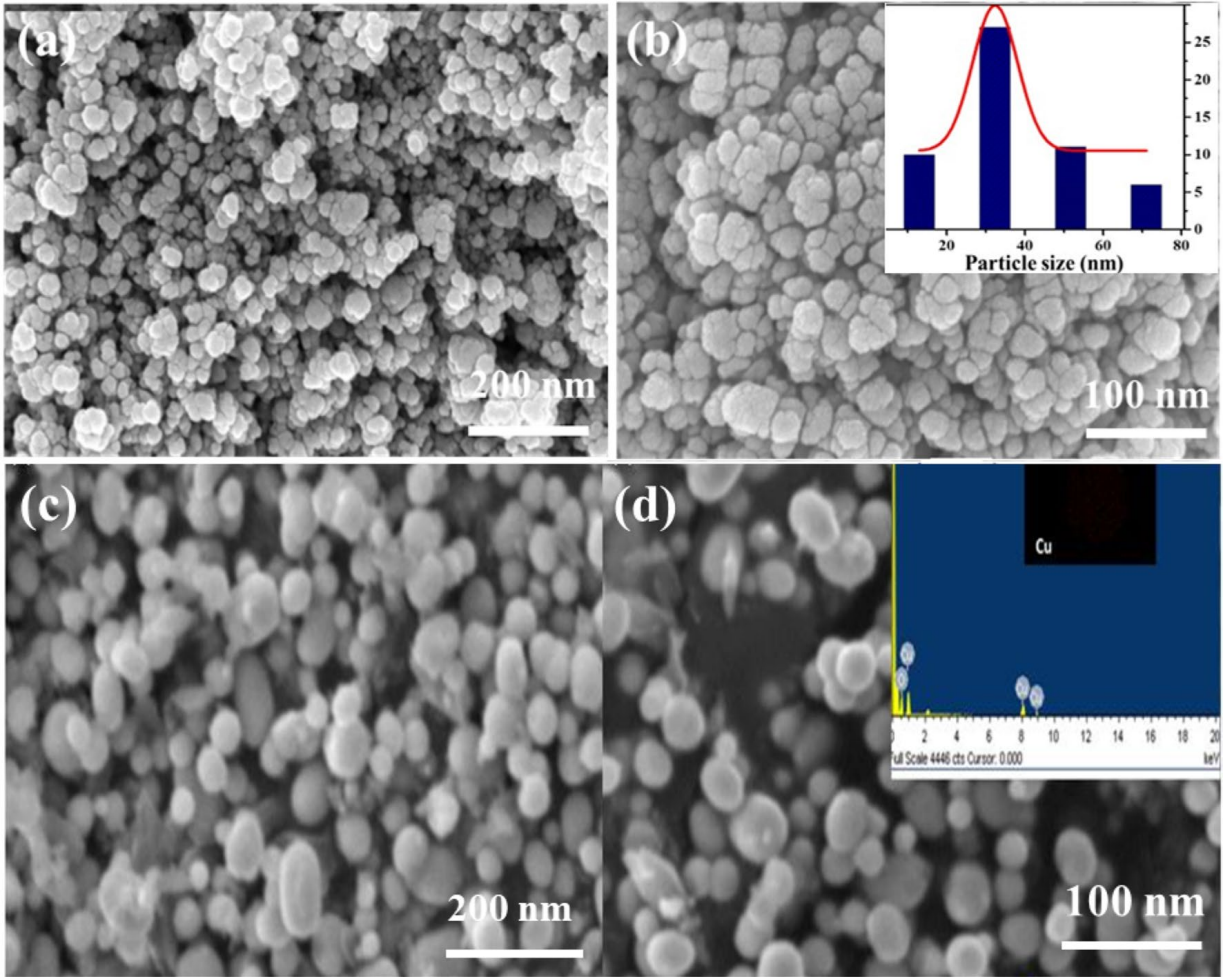
(**a,b**) SEM images of Cu_3_N (synthesized at 160 ml/min ammonia flow rate and 300 °C (inset: particle size distribution). (**c,d**) SEM image of Cu_2_O (inset: EDS spectra of Cu_2_O).

**Figure 5:**
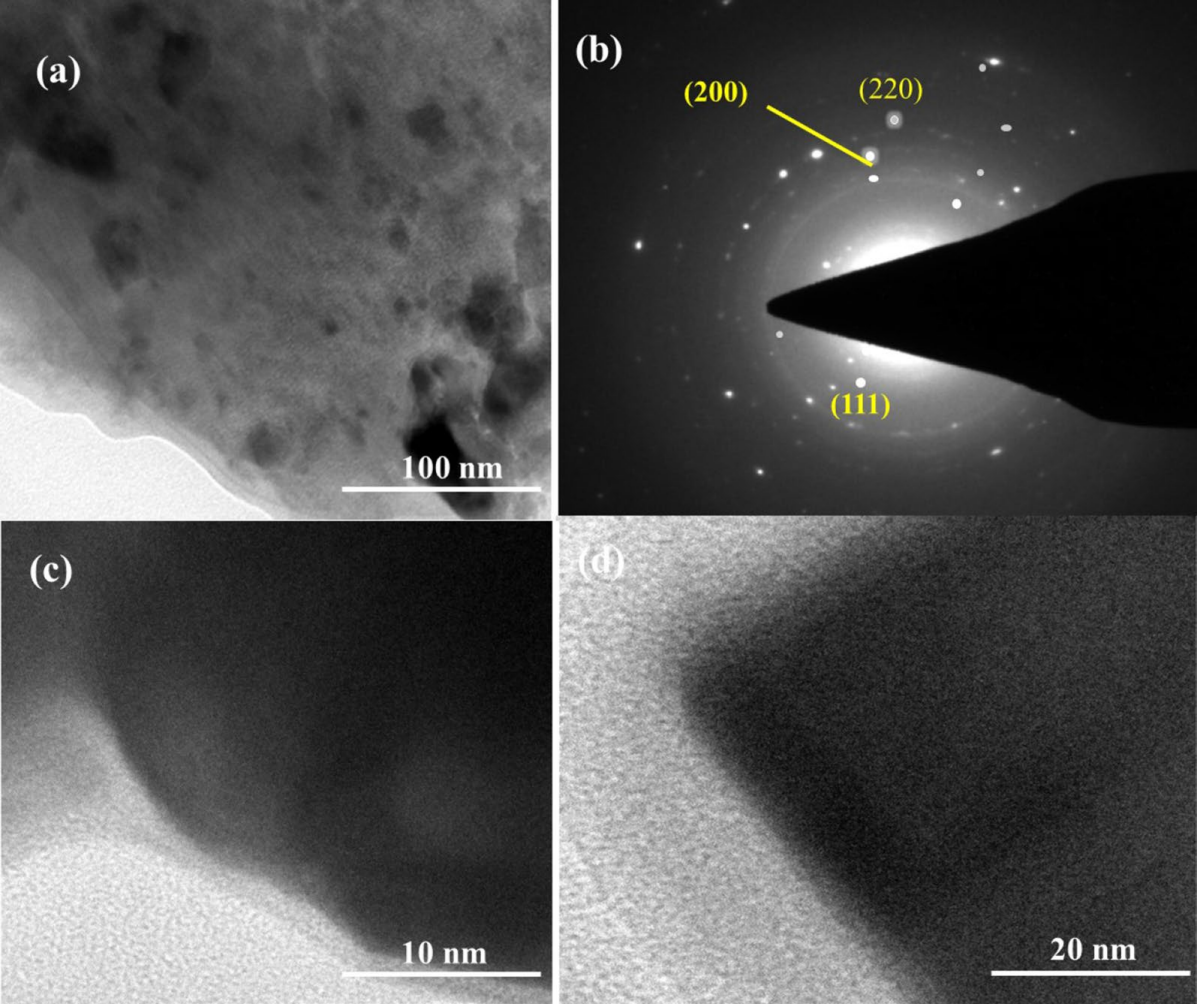
(**a–d**) TEM image and SAED pattern of Cu_3_N (synthesized at 160 ml/min ammonia flow rate and 300 °C).

**Figure 6:**
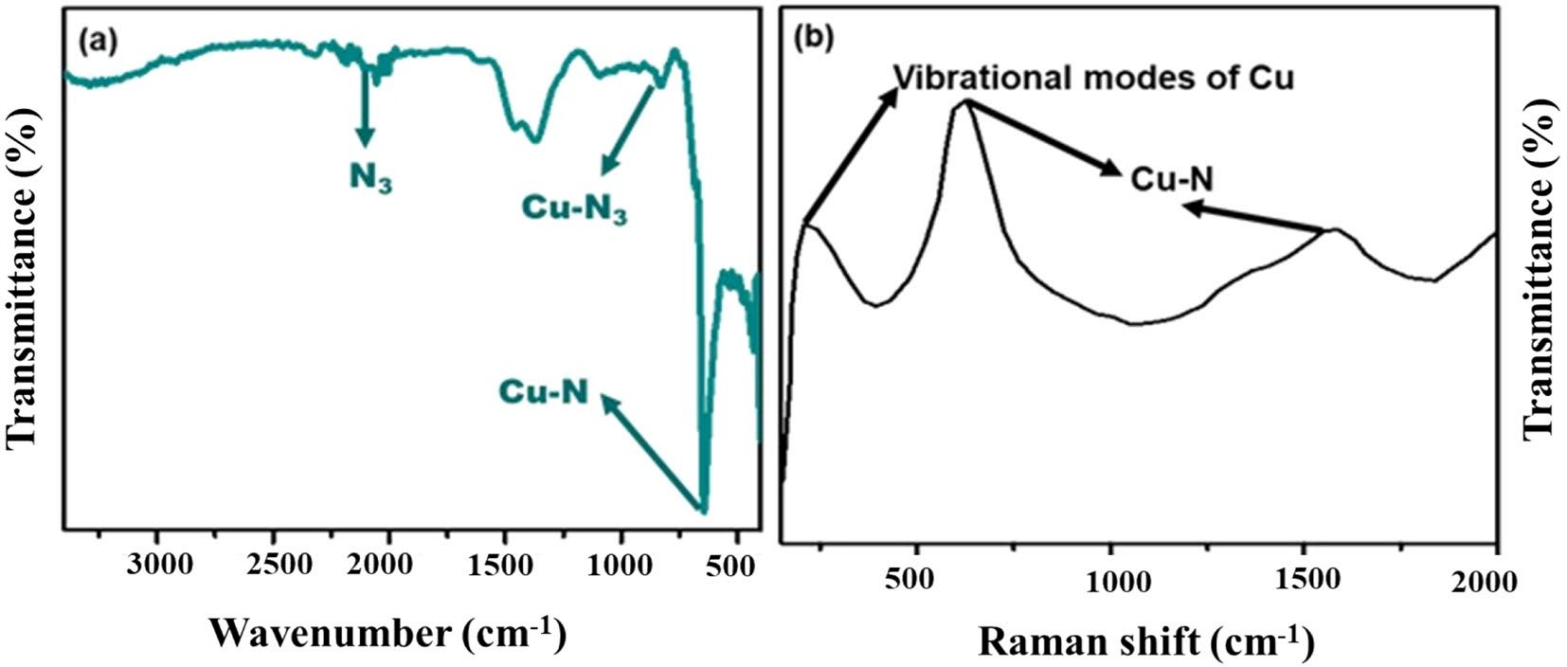
(**a**) FTIR and (**b**) Raman spectra of Cu_3_N (synthesized at 160 ml/min ammonia flow rate and 300 °C).

**Figure 7:**
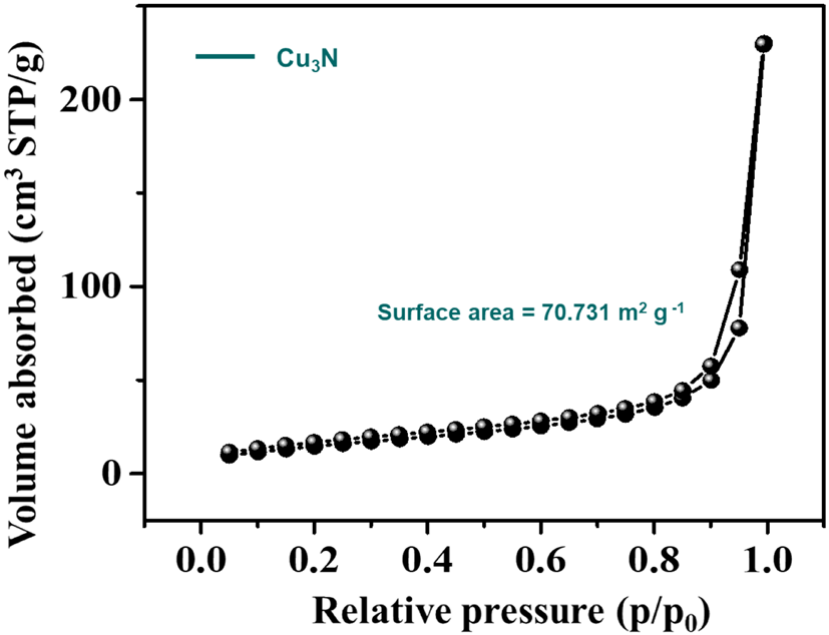
BET surface area analysis calculated from N_2_ adsorption–desorption isotherms of Cu_3_N nanoparticles (synthesized at 160 ml/min ammonia flow rate and 300 °C).

**Figure 8:**
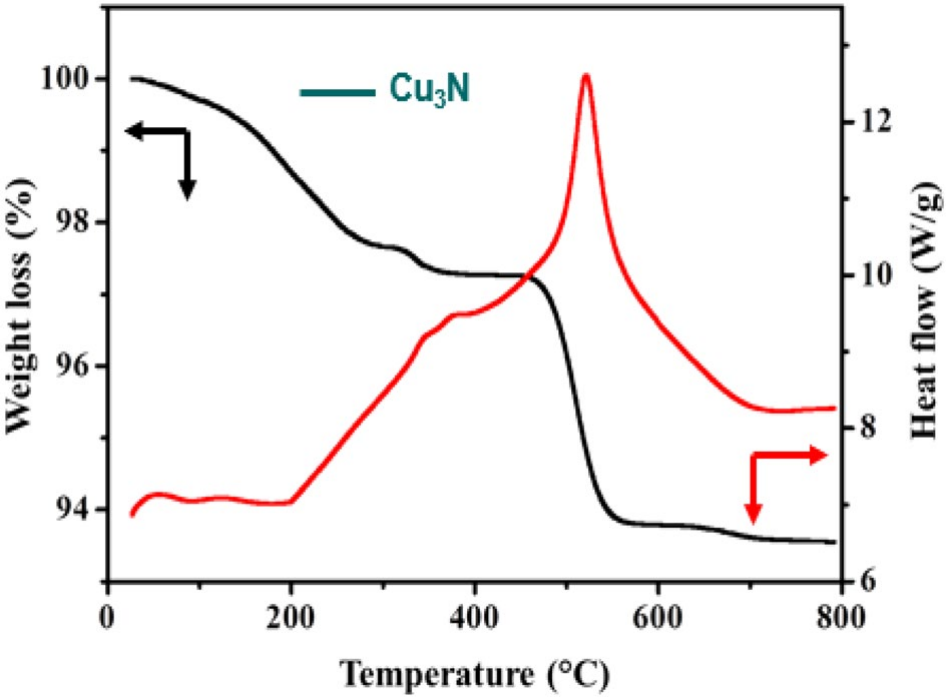
TGA/DSC analysis of the synthesized Cu_3_N at 160 ml/min ammonia flow rate and 300 °C.

**Figure 9:**
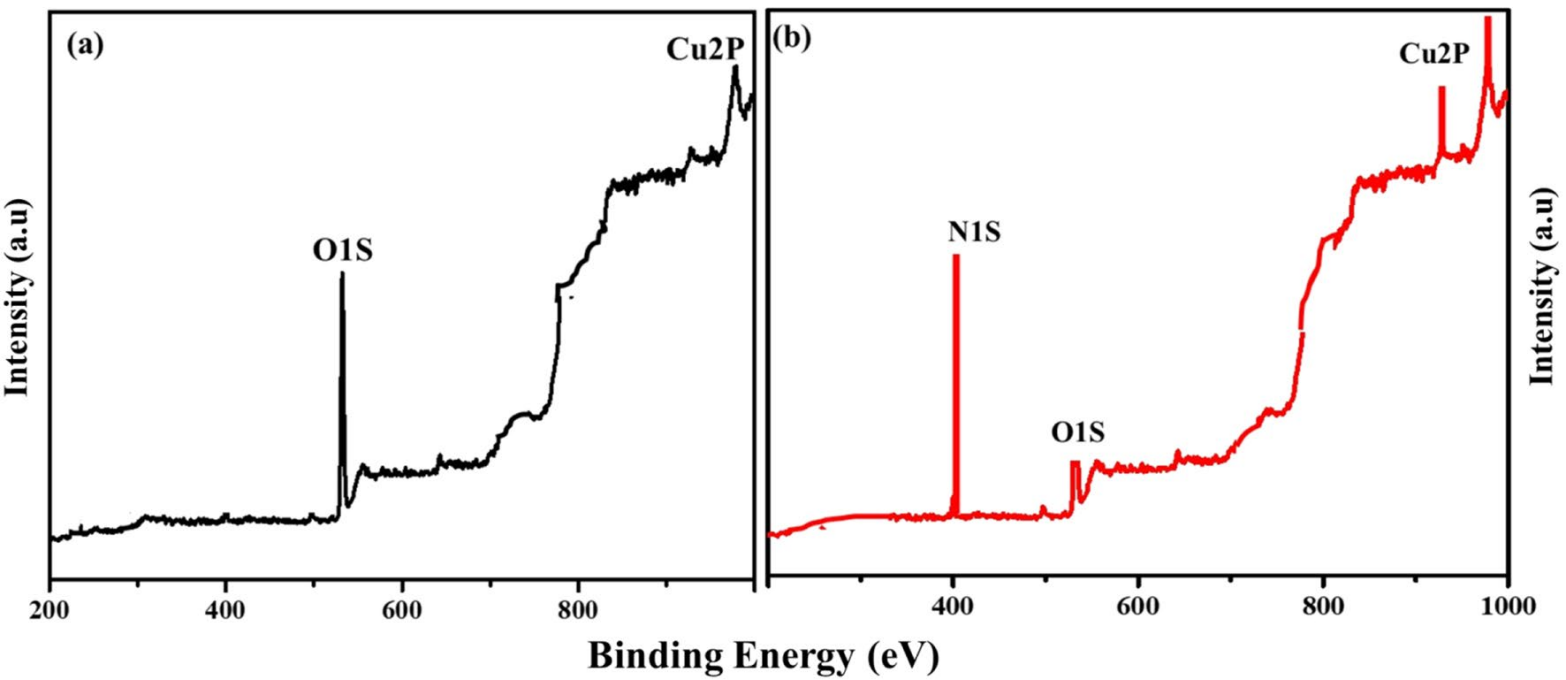
X-Ray photoelectron spectroscopy analysis of (**a**) Cu_2_O, (**b**) Cu_3_N (synthesized at 160 ml/min ammonia flow rate and 300 °C.

**Figure 10:**
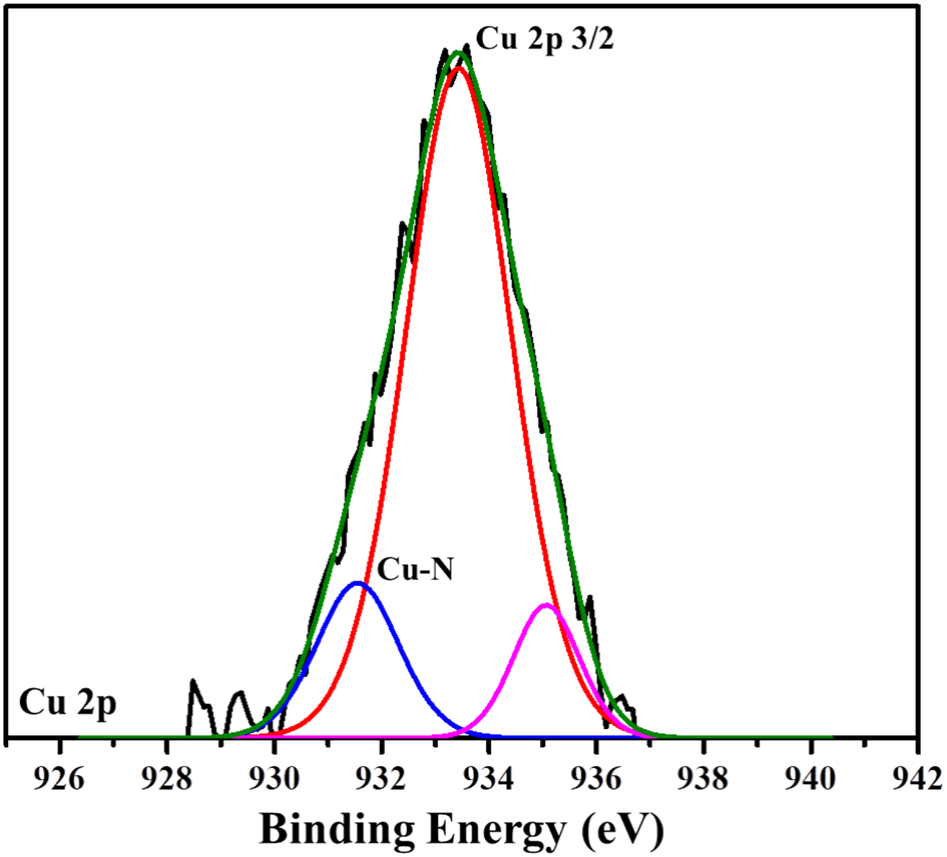
Deconvoluted spectra of Cu2p of Cu_3_N.

The nature of crystallinity and phase structure of the synthesized cuprous oxide (Cu_2_O) nanoparticles were confirmed from XRD measurements as given Fig. 2a. In the pattern, peaks at 29.3°, 36.40°, 42.5°, 61.4°, 73.4° and 77.5° are indexed to the crystallographic planes of (110), (111), (200), (220), (311) and (222). Cu_2_O has a cubic phase with lattice constant ‘a’ being 0.4266 nm, which has oxide ions (O_2_^−^) coordinated with two cuprous ions (Cu^2+^) and exactly close to the JCPDS card number value of 5–667^36,39^. The size of Cu_2_O particles as estimated from the diffraction peaks widths using Debye Scherrer equation was approximately 15 nm. Various trial experiments were done for the preparation of Cu_3_N at various temperatures and concentration of ammonia gas. In the first trial, Cu_2_O was preheated at 250 °C in NH_3_ atm (flow rate 60 ml/min). In the XRD pattern, peaks at 23.6°, 33.5°, 35.91°, 38.87°, 41.1°, 43.62°, 47.8° and 54.0° correspond to the crystal planes of (001), (110), (111), (111), (111), (111), (002), (002) and (210) respectively. In trial-1, Cu_3_N was not found with a trace mount of CuO and residual of Cu in the material. With an enhanced temperature and flow rate of NH_3_ gas in trial-2, Cu_2_O was preheated at 300 °C in NH_3_ atm (flow rate 120 ml/min), wherein Cu_3_N was not obtained as shown in Fig. 3a,b. Finally, Cu_2_O was preheated at 300 °C in NH_3_ atm (flow rate 160 ml/min) and given in Fig. 2b. The diffraction peaks observed at 23°, 33°, 41°, 48°, 54°, 59°, 69° and 74° corresponds to the crystal plane (100), (110), (111), (200), (210), (211), (220) and (300) respectively, which confirm the formation of Cu_3_N nanocrystal as per the JCPDS card No. 47-1088 with a crystalline size of Cu_3_N being 12 nm. The morphology and structural features of the Cu_3_N (300 °C/160 ml/min) were analysed by scanning electron microscopy as given in Fig. 4a,b. Cu_3_N materials are nanoclustered flower like morphology with nanoflowered structure. The average particle size was calculated to be 18.8 nm and particles distribution ranged from 30 to 40 nm respectively as given in the inset of Fig. 4b and the corresponding morphology of the Cu_2_O nanoparticles is given in Fig. 4c,d. Figure 5 shows the TEM image of Cu_3_N and from the result the lattice was found to be cubic crystal and further from the SAED pattern, it could be seen that apart from the Cu_3_N pattern, a trace amount of impurities could be seen that might be due to the presence of minor amount of unreacted Cu_2_O but the proportion was very less as observed from XPS. To further investigate the functionality and molecular structure, Fourier transform infrared spectroscopy (FTIR) analysis was carried out for Cu_3_N catalyst. As shown in Fig. 6a, FTIR spectrum of Cu_3_N nanoflower exhibited prominent peaks at 652 cm^−1^, which is ascribed to the intrinsic lattice mode vibration of Cu–N. The sharp peaks at 819 cm^−1^ is assigned to the surface of Cu–N_3_ bond. The peak at 2049 cm^−1^ corresponds to the stretching vibration of N_3_ azide confirming the formation of Cu_3_N. Further Raman spectrum was conducted to examine the Cu_3_N electrocatalyst and as given in Fig. 6b, two distinct peaks at 625 cm^−1^ and 1570 cm^−1^ correspond to the stretching and bending of Cu–N bond and the peak at 218 cm^−1^ is assigned to the vibrational mode of Cu. The porosity of electrocatalyst was investigated by nitrogen adsorption–desorption isotherm to understand the accessible surface properties, as shown in Fig. 7. The Brunauer–Emmett–Teller (BET) surface area was calculated to be 70.731 m^2^/g for Cu_3_N obtained at 300 °C/160 ml/min. It shows type II adsorption isotherm and hysteresis loop has been observed, which shows mesoporous pore size structure. The cumulative pore volume was calculated to be 5.448 × 10^–2^ cc/g with a diameter pore size of 1.92 nm. This high surface area and micropores can offer efficient active sites and also promote diffusion of ions in the electrolyte to accelerate the electrochemical process of HER. Further TGA analysis was done to understand the thermal stability of the synthesized samples. The thermogravimetric analysis of the material synthesized at various temperatures under N_2_ atmosphere was done and given in Fig. 8. As observed from the figure, the TGA curves could be identified into three different weight loss regions. During the first stage, a minor weight loss occurred at a temperature ranging from 0 to 150 °C, which is related to the loss of trapped water molecules. The second stage weight loss occurring at 250 °C is associated to the removal of organic solvents present on the surface of the particle. The third stage weight loss at 400 to 550 °C is due to the thermal decomposition of Cu and N_2_. Moreover, thermogram of Cu_3_N exhibited three weight losses, which is in agreement with the previous reported Cu_3_N materials^32–35,40,41^. DSC is a very effective characterization tool for analysing the thermal properties and heat capacity of the material and the synthesized Cu_3_N material has an exothermic peak at 520 °C. To further analyse the material, XPS was taken for Cu_2_O and Cu_3_N samples (Fig. 9a,b) and from the figure, it could be observed that Cu-related peaks exhibit a symmetric shape with no satellite peak around 943 eV, ruling out the presence of Cu^2+^. In the deconvoluted XPS spectrum of Cu_3_N, Cu 2p peak at binding energy of 932.4 eV was found with a shoulder around 934 eV. The first peak around 932 eV is attributed to Cu_3_N; two other peaks around 933 eV and 934 eV are attributed to Cu 2p3/2 and Cu^2+^ respectively. The former energy is close to the reported value of Cu_3_N from the energy of Cu metal (932.1 eV; not shown), and this slight difference between Cu and Cu_3_N agrees with close binding energies of Cu^0^ and Cu^1+^ as shown in Fig. 10.

### Electrochemical characterization

The electrochemical HER testing was carried out in three electrode cell by using electrochemical workstation (CHI660C instrument) at ambient temperature. Platinum wire, Ag/AgCl electrode was used as counter and reference electrodes respectively. The catalyst coated glassy carbon electrode was used as working electrode in 1 M NaOH alkaline solution as electrolyte for HER. All the potentials were measured with reference to Ag/AgCl (aq.) electrode and the same was calibrated to the potential versus reversible hydrogen electrode (RHE), in accordance with the equation.

$$E Vs.RHE={E}_{vs}(Ag/AgCl)+0.059\, pH+0.199 \left(V\right).$$

**Figure 11:**
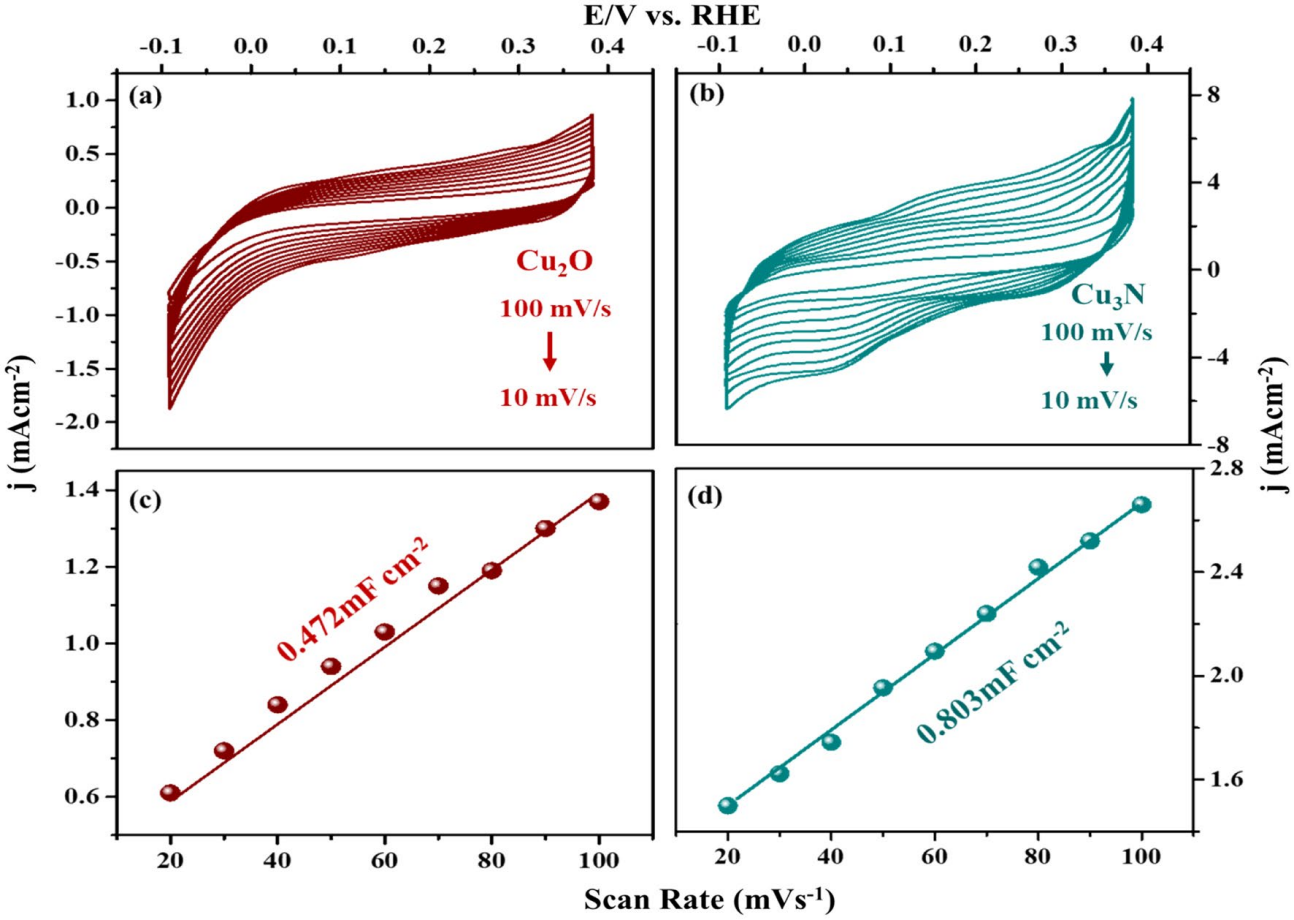
Cyclic voltametric analysis at various scan rates from − 0.1 to 0.4 V vs. Ag/AgCl (**a**) Cu_2_O, (**b**) Cu_3_N. Cdl calculations of (**c**) Cu_2_O, (**d**) Cu_3_N.

**Figure 12:**
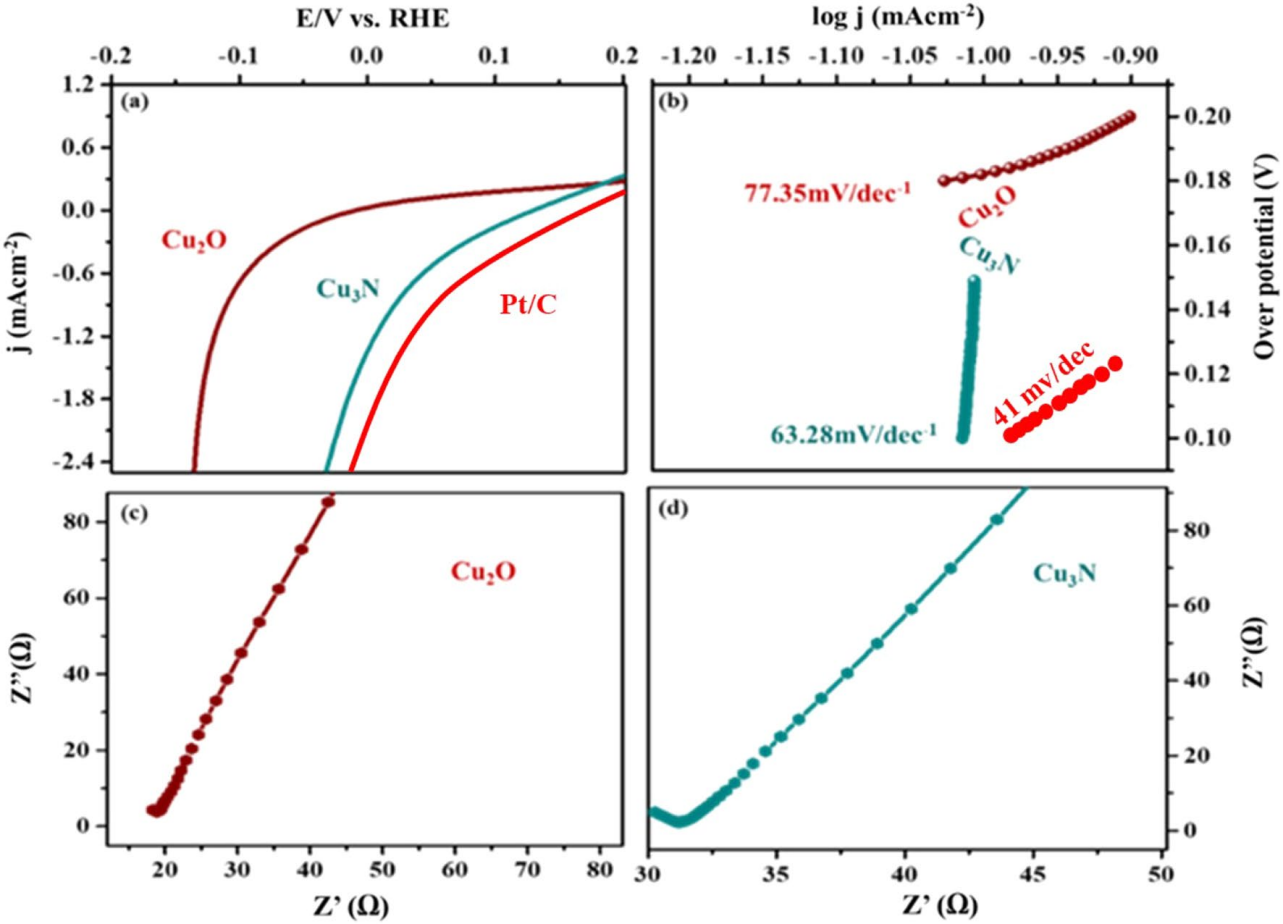
(**a**) Steady state polarization plot of Cu_3_N (synthesized at 160 ml/min ammonia flow rate and 300 °C), Cu_2_O and Pt/C. (**b**) Tafel plot in 1 M NaOH (**c,d**) Electrochemical Impedance spectroscopy (**c**) Cu_2_O, (**d**) Cu_3_N (synthesized at 160 ml/min ammonia flow rate and 300 °C.

The electrocatalytic activities of Cu_2_O and Cu_3_N towards HER were investigated by cyclic voltammetry at various scan rates (10 mVs^−1^ to 100 mVs^−1^) in non-faradic current region to evaluate the manifest of electrochemical double layer capacitance (C_dl_). HER polarization current was recorded at 2 mVs^−1^ to determine the onset potential, overpotential, Tafel slope and current density. To improve the electrocatalytic performance of Cu_2_O and Cu_3_N materials in basic medium towards HER, the electron transport and electrochemical surface area are compared (Fig. 11a–d)^38,41^. Thus, ECSA of the catalyst could be directly reflected from the double layer capacitance (cdl) as estimated from the cyclic voltammetry (CV) curves vs. scan rate (10 mVs^−1^ to 100 mVs^−1^). By following McCrory’s theory, the capacitance from EDLC was calculated^38,42^ and the double layer capacitance value calculated for Cu_2_O and Cu_3_N was calculated to be 0.472 mF cm^−2^ and 0.803 mF cm^−2^ in 1 M NaOH. This could be reflected in the higher electrocatalytic activity due to large C_dl_ value. The results indicated that Cu_3_N has higher electrocatalytic activity than Cu_2_O, because Cu_3_N materials have higher electron transfer and conductivity properties. To elucidate the possible kinetic reaction of hydrogen evolution reaction, the involvement of Cu_3_N and Cu_2_O in the reaction is explored using steady state polarization. The linear sweep voltammetry (LSV) curve was recorded at a potential window of − 0.2 V to 0.2 V at a scan rate of 2 mV in 1 M NaOH alkaline medium. In Fig. 12a at the onset potential at 10 mA cm^−2^ for Cu_3_N and Cu_2_O catalyst, it can be seen that Cu_3_N nanostructure exhibits a remarkable electrocatalytic activity towards HER with onset potential of 0.085 V for Cu_3_N lower than that Cu_2_O (0.035 V). Thus, results indicated that high surface area and favourable electrical conductivity of Cu_3_N nanoflower promotes accessible active sites and fast electron transfer than Cu_2_O nanoparticles. Further the enhanced electrochemical HER activity can be illustrated by comparing the Tafel slope. The Tafel plots are directly measured from LSV cures (overpotential vs. Log j) graph as given in Fig. 12b and these plots are used for the quantitative analysis of kinetics reaction of HER. Tafel plots were fitted in the linear potion of the Tafel equation as η = a + b log J: where J is current density, η is overpotential, b Tafelslope. The Tafel slope was calculated to be 63.28 mV/decade at an overpotential of 149.18 mV for Cu_3_N smaller than Cu_2_O nanoparticles (77.25 mV/decade and 200.6 mV). Here in numerous Cu^3+^ species was formed at Cu_3_N material during electrochemical process, which might be regarded as active sites for enhancing the electrical conductivity of Cu_3_N nanoflower beneficial for boosting the HER performance. The kinetics reaction of HER was analysed by Tafel plot. The pathway of kinetics for the conversion of (H^+^ to H_2_) in basic medium in general follows three mechanism viz. Volmer, Heyvosky and Tafel reaction. Volmer is the proton discharge electrosorption (Eq. (8)), electrochemical desorption is the Heyvosky reaction (Eq. (9)) and last step Tafel indicates the recombination of two surface-absorbed H_2_ atom (Eq. (10)).

8 $$M+{H}_{3 }{O}^{+}+{e}^{- }\to M{H}_{ads}+{H}_{2}O-\left(b \sim 120\, \text{mV}\right),$$

9 $${MH}_{ads}+{MH}_{ads}\to M+{H}_{2}-\left(b \sim 40\, \text{mV}\right),$$

10 $${MH}_{ads }+{H}_{3}{O}^{+}+{e}^{-}\to 2M+{H}_{2 }+{H}_{2 }+{H}_{2 }O-\left(b \sim 30\, \text{mV}\right),$$

where MH_ads_ represent the absorbed H_2_ atom over the surface of the metal and M represents the catalytically active free sites for HER. The Tafel slope was calculated to be 63.28 mVdec^−1^ and 77.25 mVdec^−1^ for Cu_3_N and Cu_2_O associated to Volmer–Heyvosky mechanism for the hydrogen evolution. The extrapolation of Tafel plot gives the exchange current density, which was calculated to be 24.2 mA/cm^2^ and 11.3 mA/cm^2^ respectively. A comparison table of reported Cu_3_N results are discussed in Table 1. Thus, Cu_3_N materials promote electron penetration exposing active sites and mass transfer ability, which suggest the better electrocatalytic activity towards HER. The comparison of HER activity of Cu_2_O and Cu_3_N are given in Table 2.

**Table 1:** A table of comparison of the reported catalysts with the present material reported in this work.

S. no.	Electrocatalyst	Tafel slope (mV/dec)	References
1	Cu_3_N-Copper foam	168	^38^
2	Cu_3_N-Nickel Foam	122.06	^43^
3	Cu_3_N-Cu_3_P-Nickel foam	69	^44^
4	Cu_3_N-PdO	122.83	^45^
5	Cu_3_N-NF	122	^46^
6	Cu_2_O-C_3_N_4_	55.0	^47^
7	Cu3P-CoP nanowire	96	^48^
8	Cu_2_O	77.35	This work
9	Cu_3_N	63.28	This work

**Table 2:** Tafel slope and exchange current density for HER with Cu_3_N electrode.

Electro-catalyst	Tafel slope (b mV/decade)	Log J_o_ (mA/cm^−2^)	α value	Overpotential (mV)
Cu_3_N	63.28	24.2	0.92	149.18
Cu_2_O	77.45	11.3	0.76	200.6

Electrochemical impedance spectroscopy (EIS) measurements were further done to analyse the interfacial properties of the as obtained electrocatalyst. As given in Fig. 12c,d, the semicircle in the high frequency area of the Nyquist plot was ascribed to the charge transfer resistance (Rct) and higher value of Rct denotes slow reaction rate and lower value of Rct denotes faster reaction rate.

**Figure 13:**
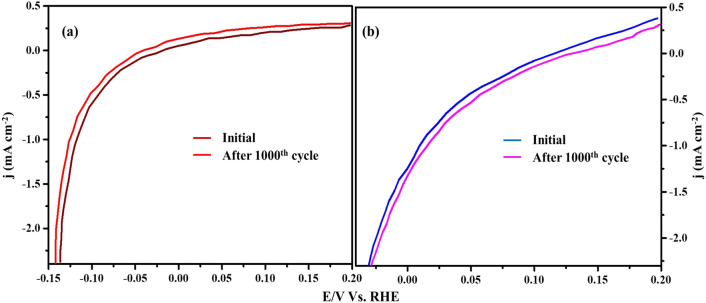
Cyclic stability tests of (**a**) Cu_2_O, (**b**) Cu_3_N (synthesized at 160 ml/min ammonia flow rate and 300 °C) tested using cyclic voltammetry at a scan rate of 2 mV/s vs. Ag/AgCl.

**Figure 14:**
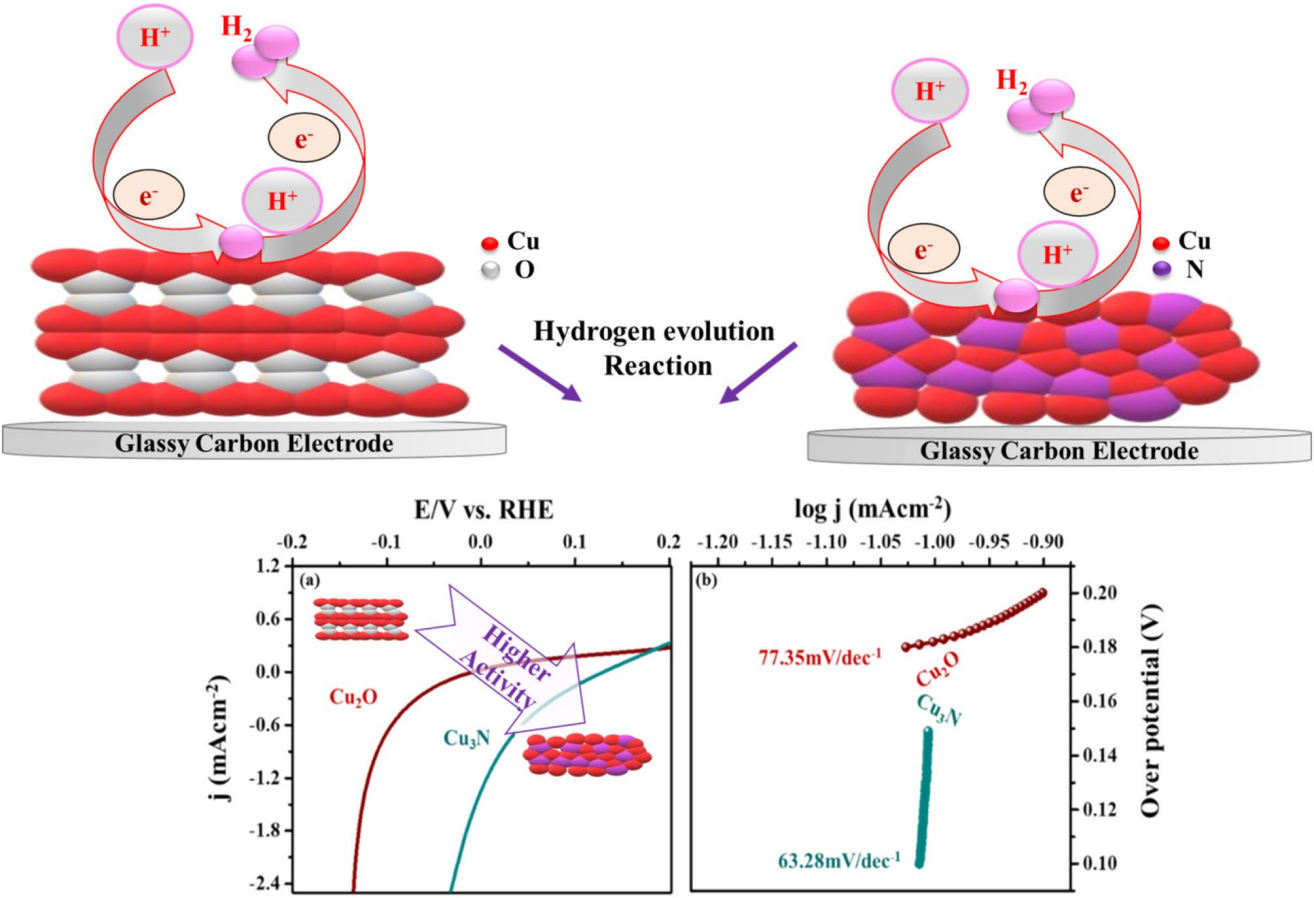
A schematic on the activity of HER using Cu_2_O and Cu_3_N electrocatalysts.

The cyclic stability test was conducted using linear sweep voltammetry from − 0.2 to 0.2 V and from the result, it was observed that the stability of Cu_3_N was good compared to the Cu_2_O as given in Fig. 13a,b. Overall results show that the synthesized Cu_3_N is an effective catalyst for electrochemical HER (Fig. 14).

## Conclusion

In summary, Cu_3_N was synthesized successfully from nitridation of Cu_2_O nanoparticles. The electrochemical hydrogen evolution reaction was carried out using Cu_3_N in alkaline medium in 1 M NaOH. By using Cu_3_N as electrocatalyst, a low Tafel slope of 63.28 mV/decade with a low overpotential of 149.18 mV was observed, which follows Volmer–Heyrovsky reaction mechanism. Thus overall results show that the catalyst has good electrocatalytic activity for HER thus making it a potential candidate for cost effective catalysts in electrochemical hydrogen production.
